# How Soundtracks Shape What We See: Analyzing the Influence of Music on Visual Scenes Through Self-Assessment, Eye Tracking, and Pupillometry

**DOI:** 10.3389/fpsyg.2020.02242

**Published:** 2020-10-07

**Authors:** Alessandro Ansani, Marco Marini, Francesca D’Errico, Isabella Poggi

**Affiliations:** ^1^Cosmic Lab, Department of Philosophy, Communication, and Performing Arts, Roma Tre University, Rome, Italy; ^2^Department of Psychology, Sapienza University of Rome, Rome, Italy; ^3^Institute of Cognitive Sciences and Technologies, Rome, Italy; ^4^Department of Education Science, Psychology, Communication Science, University of Bari Aldo Moro, Bari, Italy

**Keywords:** soundtrack, film music, audiovisual, interpretation, empathy, eye tracking, pupillometry, environment perception

## Abstract

This article presents two studies that deepen the theme of how soundtracks shape our interpretation of audiovisuals. Embracing a multivariate perspective, Study 1 (*N* = 118) demonstrated, through an online between-subjects experiment, that two different music scores (melancholic vs. anxious) deeply affected the interpretations of an unknown movie scene in terms of empathy felt toward the main character, impressions of his personality, plot anticipations, and perception of the environment of the scene. With the melancholic music, participants felt empathy toward the character, viewing him as more agreeable and introverted, more oriented to memories than to decisions, while perceiving the environment as cozier. An almost opposite pattern emerged with the anxious music. In Study 2 (*N* = 92), we replicated the experiment in our lab but with the addition of eye-tracking and pupillometric measurements. Results of Study 1 were largely replicated; moreover, we proved that the anxious score, by increasing the participants’ vigilance and state of alert (wider pupil dilation), favored greater attention to minor details, as in the case of another character who was very hard to be noticed (more time spent on his figure). Results highlight the pervasive nature of the influence of music within the process of interpretation of visual scenes.

## Introduction

The influence of music on human behavior has been studied since the dawn of time. Although a vast amount of studies analyzed the influence on several kinds of performances, among which physical tasks (Edworthy and Waring, 2006), work performance (Lesiuk, 2005), text and verbal memory (Taylor and Dewhurst, 2017), and learning (Lehmann and Seufert, 2017), the vast majority of the studies, starting from the 1980s, focused on marketing, shopping, and advertising (Bruner, 1990). Nowadays, this tradition continues, although several modifications have been made within the experimental paradigms to involve new contemporary scenarios such as online shopping, website atmospherics, and driving game performance (Brodsky, 2001).

Another flourishing tradition has started to bloom in the last two decades on the psychology of music in gambling environments: numerous researchers have deepened the influence of music on gambling, virtual roulette, ultimatum game, casino environment, and lottery (Dixon et al., 2007).

Concerning aspects in the domain of affect, a lot have been written about the processes through which music is useful for personal enhancement (Brown and Theorell, 2006), being able to express and induce moods and emotions (Västfjäll, 2001). An increasing number of studies measure plausible behavioral changes in dependence on the listening of music pieces evoking or inducing different emotions. Several social- and moral-related domains have been explored: facial emotion recognition (Woloszyn and Ewert, 2012); awareness, acceptance, and recall of unethical messages (Ziv et al., 2012); moral judgment and prosocial behavioral intentions (Ansani et al., 2019; Steffens, 2020); and compliance with requests to harm a third person (Ziv, 2015).

## Induction vs. Background Music

Among studies on music influence, we can distinguish two categories, depending on their exploiting the musical stimulus either before the task or during the task. We call the former method *induction* and the latter *background music*. This premise is paramount since the underlying mental processes that preside over these two experimental situations might be substantially different: according to a previous study (Pisciottano, 2019), in the case of induction music, the participant feels the emotion him/herself, while in the background case, s/he attributes the emotion to the scene character.

Our study focuses on soundtracks, that is musical stimuli administered as background. Background music implies a parallel and multimodal processing, which can vary among music/unrelated, music/related, and music/visual tasks. In general, depending on the task, music may have either positive, integrative, or detrimental effects.

### Detrimental Effects: Music as a Source of Distraction

Hearing music while performing an experimental task may be distracting, being a secondary source of information. Indeed, according to Kahneman’s (1973) Cognitive-Capacity Model, “there is a general limit on man’s capacity to perform mental work. […] this limited capacity can be allocated with considerable freedom among concurrent activities. […] the ability to perform several mental activities concurrently depends, at least in part, on the effort which each of these activities demands when performed in isolation. The driver who interrupts a conversation to make a turn is an example” (Kahneman, 1973, p. 9).

Not necessarily does an effortful workload exclude a mood effect—several researchers who use music during the task (e.g., Au et al., 2003) provide accounts in terms of mood, but the parallel elaboration often implies the emergence of other phenomena. In their meta-analysis, Kämpfe et al. (2011) conclude that background music has detrimental effects on several memory-related tasks and produces decreases in reading performance, being a source of distraction during cognitive tasks *per se* (Furnham and Strbac, 2002; Kallinen, 2002; Salvucci et al., 2007) or depending on its tempo (Mentzoni et al., 2014; Nguyen and Grahn, 2017; Israel et al., 2019) or its volume (Noseworthy and Finlay, 2009).

#### Integrative Effects

When it comes to music in audiovisuals, things become more complicated. In this case too music can be a distractor, leading, for instance, to a reduced recall of the ads’ messages (Fraser and Bradford, 2013). Nevertheless, the effects of music are overall integrative: on the one hand, the human mind expects music to exhibit some sort of synchrony (Rogers and Gibson, 2012) and, most of all, congruity to what is stated and depicted (i.e., visual information) by the main message, whether movies or advertising (Bolivar et al., 1994; Boltz, 2004; North, 2004; Oakes, 2007; Herget et al., 2018), as stated by the Congruence-Association Model by Cohen (2013). On the other hand, through the mood communicated, music can convey semantic and content-related information by activating specific schemas: cognitive structures developed through experience that represent “knowledge about concepts or types of stimuli, their attributes, and the relations among those attributes” (Shevy, 2007, p. 59). Such schemas, in turn, influence the building of a mood-coherent audiovisual narrative.

## Soundtrack and Interpretation

As already implied by Hoeckner et al. (2011), background music provides an interpretive framework for the audiovisuals (for a more detailed analysis of several cognitive frameworks of soundtracks, see Branigan, 2010); moreover, it can be seen as a second source of emotion besides the film itself (Cohen, 2001): it shapes audience’s understanding not only of a character’s actions, emotions, and intentions (Marshall and Cohen, 1988; Tan et al., 2007), by framing “visual meanings” (Nelson and Boynton, 1997), but also of characters’ moral judgments (Steffens, 2020), general evaluations (Shevy, 2007), and plot anticipations (Bullerjahn and Güldenring, 1994; Vitouch, 2001; Shevy, 2007) or by generating expectations (Killmeier and Christiansen, 2004). This is well-known to any director and soundtrack composer: “the music in a film may be original or not, but what matters most, from a textual and communicative point of view, is the relationship established between the music, and the script, and the photography, and how they all add up and combine with each other, so that viewers can interpret them in a certain way” (Zabalbeascoa, 2008, p. 24). Several scholars claim in fact the existence of proper semiotics of music for film and TV (Tagg, 2013).

Tagg (2006) let their subjects listen to 10 title themes for film or television, asking them “to write down what they thought might be happening on the screen along with each tune. The results were collected and reduced to single concepts;” the authors called these visual–verbal associations (VVAs). Surprisingly enough, they found some of the themes to be strongly connected with male figures and some other with female figures; moreover, masculine themes were associated with concepts like Western, fast, detective, robbery, concrete, business, traffic, shooting, and planning, whereas feminine themes were associated with love, sad, parting, destiny, tragic, death, sentimental, sitting, France, and harmonious. Along the same line, Huron (1989) claims music to be a “very effective non-verbal identifier” and thus useful for targeting certain demographic and social groups as well as determining a character’s ethos. Despite such encouraging preliminary results, only a few studies focused on the different interpretations of audiovisuals that music may foster by experimentally manipulating it.

Iwamiya (1997) studied the effect of listening to different music on the impression obtained from landscapes viewed from a car, showing that they were more pleasant when music was played as opposed to silence, and the ratings of pleasantness were highest when relaxing music was on.

Boltz (2001) analyzed the interpretations of three ambiguous clips in positive music, negative music, and no music conditions. A negative rating was connected with extreme violence and death, while the highest rating was given when the interpretation was about very happy outcomes. Coherently with her hypothesis, compared to the no music condition, the interpretations of all three clips were positive in the presence of positive music and negative in the other case. Furthermore, assumptions about the main character’s personality were measured: in the positive condition, he was considered as caring, loving, and playful, while in the negative condition, the most significant adjectives were deranged, manipulative, and mysterious.

Ziv and Goshen (2006) obtained the same results with 5- to 6-year-old children. Using the first 21 bars of the melody of Chopin’s Mazurka op. 68 n. 2 in A minor as sad music and a modified version of the same piece (transposed in C major and played faster) as happy music, the authors showed that children’s interpretations were significantly affected by the background music: sadder in the first case and happier in the second.

Using a more ecological covert design (i.e., participants were presented with an original vs. fake score of the same film sequence), Vitouch (2001) found that “viewers’ anticipations about the further development of a sequence are systematically influenced by the underlying film music” (p. 70).

In his fascinating work, Bravo (2013) studied the effect of tonal dissonance on interpretations of the emotional content of an animated short film. He hypothesized that in the same film sequence, different levels of tonal dissonance would elicit different interpretations and expectations about the emotional content of the movie scene. The short film he used as a suitable stimulus to be interpreted was very ambiguous since it did not involve clear facts in its scenes. Bravo created two soundtracks only differing as to their degree of dissonance. Comparing the subjects’ interpretations in the consonant vs. dissonant condition, it emerged that in the latter, the main character was judged as more scared, alienated, sadder, less confident, and was thought to be trying to destroy something; the character was also believed to be more sinister than nostalgic and its story more tragic than hopeful.

Finally, Hoeckner et al. (2011) deepened the interesting question about how viewers relate to movie characters in correspondence of different music and how their sense of empathy is shaped by two soundtracks: thriller music and melodrama music. They found that “compared to melodramatic music, thriller music significantly lowered likability and certainty about characters’ thoughts,” while “melodramatic music increased love attributions and lowered fear attributions.” Moreover, for the first time, they introduced the theme of empathy into the debate and, although not directly assessing its level through a specific scale, demonstrated that “musical schemas used in underscoring modulate viewers’ theory of mind and emotional contagion in response to screen characters, thus providing antecedents for empathic accuracy and empathic concern.”

All of these studies are overviewed in Herget’s (2019) comprehensive review on music’s potential to convey meaning in film; she concludes her work by underlining some weak points that should be overcome to improve such domain of research:

(1) Research on this issue is sparse. This results in experiments each analyzing a single psychological construct.
(2) Complex psychological constructs such as sympathy and empathy toward media protagonists could and should be investigated through all the available established measuring instruments (Herget, 2019).
(3) There are hardly any ecologically valid investigations carried out in natural contexts such as during a visit to the cinema, a television evening with the whole family, or alone in a young person’s room (Bullerjahn, 2005);
(4) Most research designs are too complicated and extensive.
(5) Within-subjects designs risk drawing the participants’ attention to the musical manipulation (Tan et al., 2007).

Since we strongly agree with the bulk of Herget’s criticism, our aim in this work is to investigate the effects of music in the interpretation of visual scenes by specifically addressing these demands. In our Study 1 below, we intend to:

(1) provide a global view of the influence of background music on scene interpretation by examining various psychological constructs, such as empathy, affective states, and perceived personality traits;
(2) accurately measure such constructs by relying on available established measures and tools;
(3) improve ecological validity by running an online study to be directly done from the participants’ homes on laptops and other devices;
(4) reduce the number of factors to have better control and lower the number of experimental subjects required;
(5) plan a between-subjects design to prevent the subjects’ awareness of the manipulation.

Moreover, in Study 2, we employ the eye-tracking methodology to investigate the influence of music on a scene interpretation also from a physiological perspective.

## Study 1: Online Survey

As stated above, the literature on the interpretation of audiovisuals has proved the ability of music to convey meanings through associations (Cohen, 2013) and activation of cognitive schemas (Boltz, 2001). In our study, we consider interpretation in a multidimensional fashion as a global process involving several interconnected cognitive operations: attribution of emotions, personality traits, thoughts or behavioral intentions to the characters on the scene, empathy toward them, and perception of the surrounding environment.

### Research Questions

Our aim is to investigate how in a visual scene the following dependent variables are affected by background music:

(1) empathy toward the character.
(2) affective states attributed to the character.
(3) impressions of the character’s personality.
(4) plot anticipation.
(5) environment perception.

### Method: Rationale and Recruitment

**FIGURE 1:**
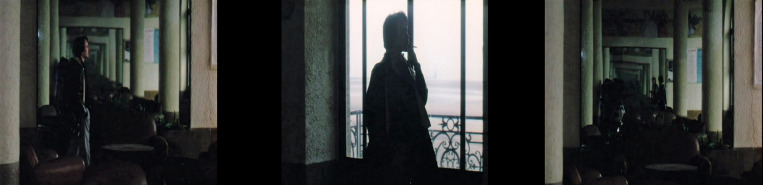
Illustration of three representative frames of the scene.

We designed a between-subjects experiment (*N* = 118–44, female; age = 37 ± 11, see Table 1 for gender and age distribution) in which participants watched a scene (01′ 55′′) from an almost unknown short movie (Duras, 1981) (Figure 1): an emotionally neutral male character slowly walks toward some large windows in a lonesome building, with the seaside in the distance. He walks, looks outside, stops, and moves out of the frame.

**TABLE 1:** Gender and age distribution (mean age ± SD).

		Controls	Evans	Rachmaninov	Total
Gender	M	27 (36 ± 10)	24 (37 ± 10)	23 (34 ± 13)	74 (36 ± 11)
	F	8 (44 ± 11)	13 (38 ± 13)	23 (35 ± 12)	44 (38 ± 12)
Total		35 (38 ± 10)	37 (38 ± 11)	46 (35 ± 12)	118 (37 ± 11)

Through Adobe Premiere Pro, we created three versions of the scene—the three experimental conditions—with the video accompanied respectively by a dogged and anxious orchestral piece (*The Isle of the Dead* by S. Rachmaninov), a soft, melancholic jazz solo piano (*Like Someone in Love* by B. Evans), or by ambient sound only. We chose these two pieces based on the findings of Juslin and Laukka (2004), and subsequent studies listed by Cespedes-Guevara and Eerola (2018), concerning several psychoacoustic parameters associated with emotional expression in music. The two pieces both evoke negative feelings but differ in the arousal dimension: Evans track’s mellow tone and soft intensity can be associated with delicacy, gracefulness, relaxation, and quietness (Fabian and Schubert, 2003) or with sadness and tenderness (Quinto et al., 2014). On the contrary, Rachmaninov track’s large sound level variability, rapid changes in sound level, and ascending pitch could be linked to fear (Juslin and Laukka, 2004), while its increasingly louder intensity could communicate restlessness, agitation, tension (Fabian and Schubert, 2003) or anger, fear (Scherer et al., 2015), and scariness (Eerola et al., 2013).

After viewing the scene, participants were asked how they felt toward the character and what they thought he was feeling, what kind of personality he could have, whether they thought he was remembering or planning instead, and how they perceived the environment in which the scene was set. To avoid sequence effects, the order of questions was randomized for each participant.

Aiming at a better ecological validity, to let people participate in a less detached situation than a lab, we build the procedure on Qualtrics.com. By accessing a single unreusable link,^1^ they could run the experiment directly from home on their laptops, smartphones, or tablets; recruited through Amazon Mechanical Turk, they were paid according to the standard American minimum wage: 1$ for a ∼15 min task.

### Measures and Hypotheses

We hypothesize that the narratives (hence, the interpretations) that the participants will build on the scene, influenced by the soundtrack, will be very different among them. We plan to shed light on such differences by taking a fine-grained look into some of the psychological constructs involved. Below, we describe each construct and its related measurement separately, stating our hypotheses at the bottom of each subparagraph.

In a nutshell, here is an example of two different plausible narratives:

• *Evans*: we see a sad man who walks alone in an empty building, he must be an introverted guy, we see that he’s watching outside the window, maybe he’s thinking about the past, maybe a loved one, the scene is sweet and quite gloomy.
• *Rachmaninov*: we see an ambiguous character walking in an unsettling hall, he shows a solemn gait, something bad is happening; probably he’s planning something harmful. I wouldn’t trust this man.

#### Empathy Toward the Character

To assess the participants’ empathy toward the main character, after comparing various indexes (Neumann et al., 2015), we opted for a 14-item two-factor scale by Batson et al. (1983). The scale involves 14 adjectives that describe affective states of distress (alarmed, grieved, upset, worried, disturbed, perturbed, distressed, troubled) and empathic interest (sympathetic, moved, compassionate, tender, warm, softhearted). The score obtained from the difference between empathic interest and distress should therefore be the most significant assessment of the empathic response (Batson et al., 1983; Leone et al., 2008): higher ratings correspond to higher empathic interest, while lower ratings stand for enhanced distress-like feelings.

H_1_: Evoking feelings of delicacy and tenderness, the melancholic track (Evans) will encourage empathy toward the character. On the contrary, the negative feelings evoked by Rachmaninov will dampen empathy.

#### Affective State Attributed to the Character

We administered a classic 10-item Positive and Negative Affect Schedule (I-PANAS-SF) for the emotions attributed to the character. The used version was previously validated by Karim et al. (2011). Moreover, we added the item *wistful*, as we were convinced that it could have been significantly different among the conditions.

H_2__a_: The dogged and menacing track (Rachmaninov) will lead to attribute a more positive affective state; the character will appear as adamant; on the contrary, Evans track will let the participant attribute the character more negative affective states.

The reason for such a hypothesis is intuitive: in the first case, music can make one imagine an evil character, possibly determined to do something harmful; while in the second case, music mood will let one picture a depressed/nostalgic character, therefore with a more negative affective state.

H_2b_: Evans track will show higher scores in wistfulness as opposed to Rachmaninov’s.

#### Impressions of Personality

To measure the participants’ personality impressions (Asch, 1946) about the character, we employed a 15-items assessment of the Big Five (Lang et al., 2011) previously validated with satisfying results.

H_3_: In the light of the melancholic track, the character will be seen as more agreeable and open (i.e., very emotional) and less extroverted; on the contrary, in dependence of Rachmaninov’s track, the character will be regarded as more neurotic and conscientious (e.g., a lucid criminal) (Table 2).

**TABLE 2:** Impressions of personality (hypotheses).

Personality trait	Evans	Rachmaninov
Neuroticism	−	+
Agreeableness	+	−
Conscientiousness	−	+
Extraversion	−	+
Openness	+	−

#### Plot Anticipation: Past Perspective vs. Future Perspective

As for the plot anticipation, we simply asked the participants whether they thought that the main character was remembering the past (*past perspective*) or taking a decision (*future perspective*). It was also possible to choose both options. In the first case, several five-point Likert scales were presented about the emotions that the characters could have been feeling in relation to his memory. In the second one, other Likert scales were presented on the nature of such a decision; in particular, we asked whether it could have been a morally good, neutral, or bad action. In the event that both the options (i.e., remembering and taking a decision) were chosen, both the questions on the memory and the decision appeared.

H_4_: When viewed with Evans music in the background, the participants will think about someone who is remembering something nostalgic; with Rachmaninov, he will be seen as a planner of possibly evil deeds.

#### Environment Perception

We were interested in understanding whether a place could be seen as cozier and warmer rather than inhospitable and unpleasant in dependence of different music; therefore, we took inspiration from a study by Yamasaki et al. (2015): they analyzed the impact of music on the impressions of the environment on the three standard dimensions of emotions: activation, valence, and potency. We decided to administer a short list of five bipolar five-step Likert scales by picking only the couples of adjectives that were somehow related to the idea of coziness, so we chose four out of five from those of the valence dimension (we excluded one for reasons of redundancy), and we also added a new couple that we considered crucial: dangerous–safe.

H_5_: The melancholic track will let the environment be perceived as cozier. On the contrary, Rachmaninov will let our participants perceive an unpleasant environment.

### Preliminary Sample Data Analysis

Every online procedure has the merit of guaranteeing a significant number of participants in a few days; nevertheless, lacking in experimental control, a careful preliminary analysis is necessary. To improve the reliability of our sample, first, we added an attention check question in which a multiple-item Likert scale was presented with an explicit instruction to avoid filling it out; thus, we excluded all those participants who compiled such a scale. Second, we added a time count on the screen containing the video so to exclude all of those participants who had not watched the whole scene.

After such exclusions, our sample decreased from 309 to 118 participants. No further outliers were excluded.

### Results

For all statistical analyses, IBM SPSS 26.0 was used; violin plots were made by means of XLStat 2020 3.1.

#### Empathy Toward the Character

**FIGURE 2:**
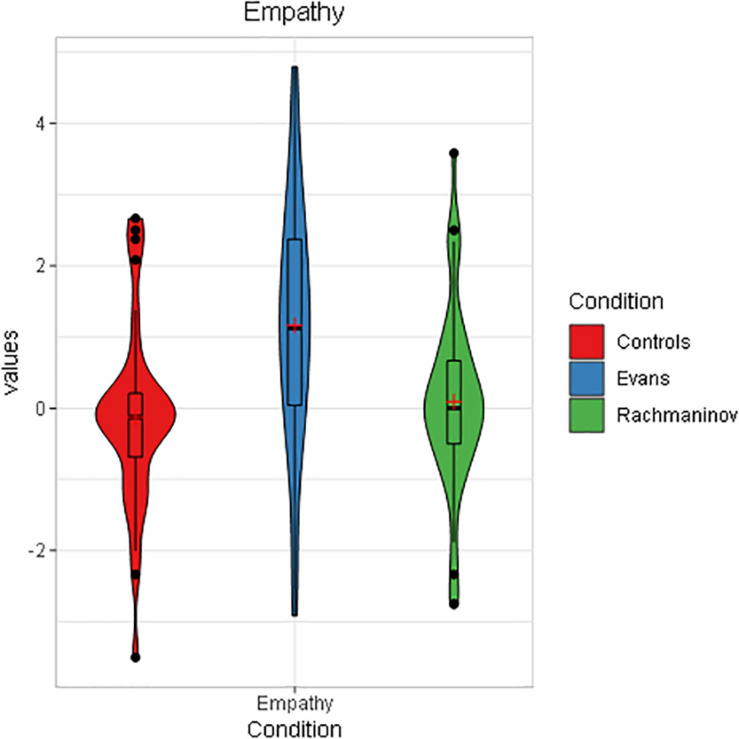
Empathy toward the character as a function of condition in Study 1 (violin plot). The boxplots within each violin represent interquartile ranges (IQRs). Red crosses indicate means, black horizontal lines indicate median, and black points are outliers. Participants in Evans condition showed significantly higher Empathy ratings.

From a one-way ANOVA, it emerged that the soundtrack significantly affected the empathy felt toward the main character (scale reliability α = 0.93), *F*(2,115) = 6.86, *p* = 0.002, $\eta_{\text{p}}^{2}$ = 0.107, (1 – β) = 0.92. The Evans group showed the highest empathy (*M* = 1.16, *SD* = 1.75), followed by the Rachmaninov group (*M* = 0.21, *SD* = 0.1.53) and controls (*M* = −0.13, *SD* = 1.29). Bonferroni corrected *post hoc* revealed that Evans group’s means were significantly different from that of the controls (*p* = 0.002) and Rachmaninov group (*p* = 0.02) (Figure 2).

We can conclude that H_1_ was verified even if we did not record the decrease in empathy in the Rachmaninov condition as opposed to controls. This event (i.e., the absence of statistically significant differences between controls and Rachmaninov group) will appear in several analyses throughout the paper; we discuss it thoroughly in Section “General Discussion.”

#### Affective State Attributed to the Character

The soundtrack did not affect the affective state (H_2a_) attributed to the character (scale reliability α = 0.85); the omnibus one-way ANOVA was not significant (*p* > 0.05). Nevertheless, the adjective *wistful* showed significance. In line with H_2__b_, the jazz melancholic track (Evans) led to higher scores in wistfulness (*M* = 3.22, *SD* = 1.39), while the Rachmaninov group (*M* = 2.43, *SD* = 1.32) and controls (*M* = 2.94, *SD* = 1.30) showed lower ratings, *F*(2,115) = 3.64, *p* = 0.029, $\eta_{\text{p}}^{2}$ = 0.060, (1 – β) = 0.66. In this case, the mean difference of the two soundtracks was significantly different (*p* = 0.029).

#### Impressions of Personality

A one-way multivariate ANOVA (MANOVA) was conducted with the five personality traits as dependent variables and the soundtrack as the independent variable. A significant multivariate main effect was found for the soundtrack, *F*(10,222) = 3.30, Wilks’ Λ = 0.76, *p* = 0.001, $\eta_{\text{p}}^{2}$ = 0.13, (1 – β) = 0.98.

The soundtrack significantly affected three out of five attributed personality traits (scale reliability α = 0.73), as in Table 3.

**TABLE 3:** ANOVA—personality traits attributed to the character.

Personality trait	*F*	*p*	$\eta_{\text{p}}^{2}$	(1 – β)
Neuroticism	0.720	0.488	0.012	0.170
Agreeableness	3.43	0.036	0.056	0.633
Conscientiousness	4.99	0.008	0.080	0.803
Extraversion	3.10	0.049	0.051	0.587
Openness	0.648	0.525	0.011	0.156

The Evans group showed the highest agreeableness toward the character (*M* = 4.51, *SD* = 1.33), followed by the Rachmaninov group (*M* = 3.98, *SD* = 1.08) and controls (*M* = 3.84, *SD* = 1.09). Subsequent Bonferroni-corrected *post hoc* comparisons revealed that Evans group’s means were significantly different from that of the controls only (*p* = 0.048).

The Rachmaninov group, on the contrary, imagined the character as more conscientious (*M* = 5.61, *SD* = 1.27) as opposed to the Evans group (*M* = 4.91, *SD* = 1.32) and controls (*M* = 4.82, *SD* = 1.16). The same *post hoc* showed that the Rachmaninov group significantly differed from both the Evans group (*p* = 0.041) and controls (*p* = 0.017).

As for extraversion, participants in the Evans group registered the lowest score, namely, imagining a shier character (*M* = 2.72, *SD* = 1.08), as opposed to the Rachmaninov group (*M* = 3.38, *SD* = 1.29) and controls (*M* = 3.20, *SD* = 1.25). *Post hoc* enlightened a significant mean difference between the two soundtracks only (*p* = 0.047).

We can conclude that H_3_ has been partially proven for three out of five personality traits.

#### Plot Anticipation: Past vs. Future Perspective

**FIGURE 3:**
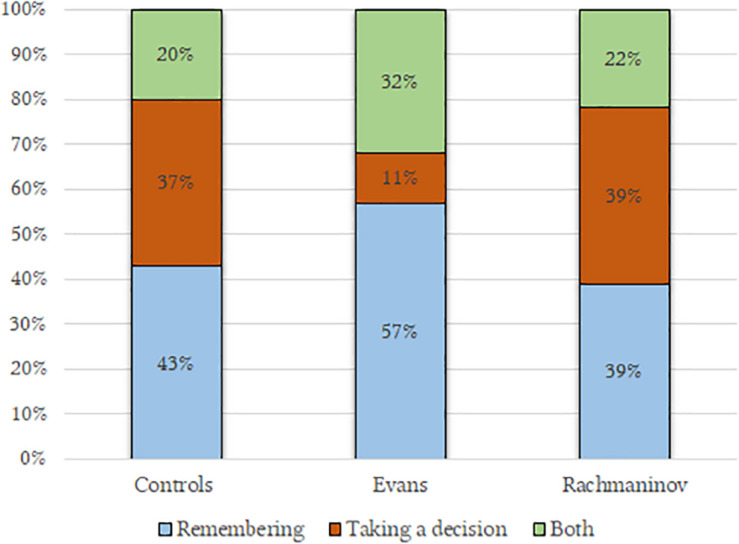
Plot anticipations as a function of the condition. Participants in Evans condition imagined the character to be remembering something; on the contrary, Rachmaninov’s track led the participants to imagine that the character was taking a decision.

As in H_4_, Evans music made the participants think that the character was above all remembering (57%) (*past perspective*), more than in the Rachmaninov group (39%), while Rachmaninov’s music let the character be seen as someone planning something (39%) (*future perspective*), more than in the Evans group (11%), c^2^(4,118) = 9.34, *p* = 0.05, η condition dependent = 0.046 (Figure 3). The controls’ pattern is comparable to one of the Rachmaninov group.

##### Emotions related to possible character’s memories

Evans’ music led to imagine slightly pleasant memories (*M* = 0.10, *SD* = 0.98) as opposed to negative memories in Rachmaninov’s track (*M* = −0.70, *SD* = 1.16) and controls (*M* = −0.23, *SD* = 1.11), *F*(2,80) = 4.26, *p* = 0.017, $\eta_{\text{p}}^{2}$ = 0.09, (1 – β) = 0.73. Subsequent Bonferroni-corrected *post hoc* comparisons revealed that the significant mean difference was the one between the two soundtracks (*p* = 0.014) (Table 8).

##### Moral nature of the decisions

In line with our hypotheses, participants who embraced the future perspective were influenced by the soundtrack in foreseeing the character’s decisions (*behavioral intentions*), *F*(2,61) = 4.87, *p* = 0.011, $\eta_{\text{p}}^{2}$ = 0.138, (1 – β) = 0.78: the Evans group thought it to be a good decision (*M* = 1.21, *SD* = 1.04) as opposed to the controls (*M* = 0.25, *SD* = 1.24) and Rachmaninov group (*M* = 0.01, *SD* = 1.35). The mean difference between the two soundtracks emerged through a Bonferroni-corrected *post hoc* analysis (*p* = 0.010) as the significative one (Table 8).

#### Environment Perception

Having reached an acceptable scale reliability (α = 0.71), to verify H_5_, we proceeded to a one-way ANOVA that showed a main effect of the condition, *F*(2,115) = 4.15, *p* = 0.02, $\eta_{\text{p}}^{2}$ = 0.067, (1 – β) = 0.72. Subsequent Bonferroni-corrected *post hoc* analysis showed the mean difference between the two soundtracks only to be significant (*p* = 0.022). As hypothesized in H_5_, the Evans group perceived the environment as cozier (*M* = 4.34, *SD* = 1.15) as opposed to the controls (*M* = 4.17, *SD* = 1.24) and Rachmaninov group (*M* = 3.67, *SD* = 0.95) (Table 8).

#### Gender Differences

In order to search for gender differences, gender was added in every analysis as the factor but showed neither main effects nor interactions.

## Study 2: Self-Assessment and Eye Tracking

Given the very satisfying results of Experiment 1, through which we proved the multifaceted influence of the soundtrack on the interpretation of a scene, we wondered whether this influence could be due to the schemas mentioned above (Boltz, 2001), whose activation could be demonstrated by eye movements; therefore, we planned to replicate the same experiment in our lab but with the addition of eye tracking.

Eye tracking has proved to be a precious technique for the analysis of several domains of cognitive science, among which were visual attention (Wedel and Pieters, 2017), cognitive workload (Kosch et al., 2018), and human interaction (Brône and Oben, 2018).

Concerning our question, we believe that different eye activities could show the activation of different cognitive schemas (Boltz, 2001), therefore configuring the (often unconscious) music perception as a proper top–down process shaping the interpretative process. To the best of our knowledge, after the seminal work of Coutrot et al. (2012), in which the presence vs. absence of sound determined different eye movements, there have been few attempts to measure eye-movement parameters in correspondence of different scenes with music, with no congruent results due to the different experimental designs and the dependent variables analyzed. Auer et al. (2012) managed to find differences in scanpaths and attention (perception of a red X in a clip with different soundtracks); similar results on attention, operationalized through the concept of spatial exploration length, have also been found by Mera and Stumpf (2014). Moreover, in a pilot experiment with a tiny sample, Wallengren and Strukelj (2015) showed an influence of film music on fixation durations in several clips; the same authors ran an improved version of the same experiment (Wallengren and Strukelj, 2018), finding marginally significant differences in the eyeblinks in dependence of different soundtracks without replicating the findings on fixation durations. In particular, they found that eyeblinks increased when film clips and music were congruent.

As for the more general analysis of dynamic stimuli such as movie scenes, not much has been done: Breeden and Hanrahan (2017) released a useful dataset for the analysis of attention in features films; in a psycho-narratology study, Kruger (2012) successfully correlated eye-tracking data with viewer constructions of the narrative of a film. Moreover, by applying a Cognitive Computational Cinematics (CCC) approach to film cognition, in two works, Smith (2013, 2014) managed to confirm filmmakers’ intuitions about the influence of motion, feature contrast, and faces on viewer attention, using a combination of eye-tracking and computer vision analyses of video content. Besides, in a recent similar work, Batten and Smith (2018) faced the same theme with a similar procedure, analyzing also gaze similarity values between audio and silent conditions. They did not find an effect of sound on gaze, but the effect of audio consisted in an earlier capture of attention and self-reported higher measures of happiness and excitement.

As a matter of fact, the viewing of a narrative scene is a complex process involving at least two intertwining components, since eye movements can be exogenous or endogenous: in the former case, they are stimulus driven and depend on the visual features of the video (bright objects, camera movements, faces); the latter are, on the contrary, linked to high-level cognitive processes, such as search tasks. We believe music to be involved in this latter case, as it properly shapes the visual scene by providing a frame through which it can be interpreted.

### Research Questions

In this study, both the hypotheses and measurements of the self-report are the same as for Study 1. Yet, we additionally hypothesized that the anxious soundtrack, increasing the participants’ arousal, could lead them to pay greater attention to the scene. To test this, a detail of the scene was exploited: a hidden cameraman appearing in the first and the last part of the scene; being set in a darkened building, the scene was overall dark, and this character appeared in the darkest part of the hall, very hard to be seen. Therefore, the only additional question in this second study asked participants whether they had seen another character aside from the main one.

### Method and Recruitment

We recruited participants (*N* = 92, 63 were female; age = 26 ± 8, see Table 4 for gender and age distribution) on a voluntary basis among students of Psychology of Communication and Cognitive science. They were symbolically rewarded with 2€ each, all of them had normal or corrected-to-normal vision. The experiment was carried out in our lab at Roma Tre University. The setting consisted of a desk with a computer and an eye-tracking device on. The average distance from the pupil to the screen was 65 cm. The screen resolution was 1,920 × 1,080 (48 cm × 27 cm).

**TABLE 4:** Gender and age distribution (mean age ± SD).

		Controls	Evans	Rachmaninov	Total
Gender	M	9 (23 ± 3)	10 (27 ± 10)	10 (27 ± 7)	29 (26 ± 8)
	F	21 (30 ± 11)	21 (24 ± 4)	21 (24 ± 6)	63 (26 ± 8)
Total		30 (28 ± 10)	31 (25 ± 7)	31 (25 ± 7)	92 (26 ± 8)

The audio (stereo—192 kHz, 24 bit) was transmitted in the room through two speakers (JBL professional LSR305 First-Generation 5′′ two-way powered studio monitor). To avoid intensity effects, the volumes of the two tracks were normalized using a Loudness, K-weighted, relative to Full Scale (LKFS) (Grimm et al., 2010).

The lighting of the room was standardized, with no sunlight entering. To improve ecological validity and to avoid a possible Hawthorne effect, during the entire experimental procedure, the operator was in the adjoining room with no possibility to see the participant. Participants were told to call for help at any time needed.

Before the task, a calibration of the eye tracker was performed for each participant. In this procedure, the participant had to gaze-follow a circle (2 cm diameter) that moved throughout the screen in a Z shape. At the end, the software reported the quality of the calibration. Only subjects with good or excellent quality were tested. When the quality was poor, a new calibration was run until reaching a satisfying quality. Besides, an operator (Author 1 or 2) checked during the whole task whether the gaze paths were reasonable (i.e., absence of any critical artifacts).

In the following, we first overview the results of Study 2 that replicated Study 1—empathy toward the character (*Empathy Toward the Character*), attribution of affective states (*Affective State Attributed to the Character*), impressions of personality (*Impressions of Personality*), plot anticipation (*Plot Anticipation: Past vs. Future Perspective*), and environment perception (*Environment Perception*); then, we detail hypotheses, measurements, and results of the eye-tracking part of Study 2 (*Eye Tracking and Pupillometry: Methodology, Metrics, and Hypotheses*); and finally, we overview the results of the survey and eye-tracking taken together (*Aggregation of Self-Reports and Eye-Tracking Data*).

### Results of the Study 2 Survey

#### Empathy Toward the Character

**FIGURE 4:**
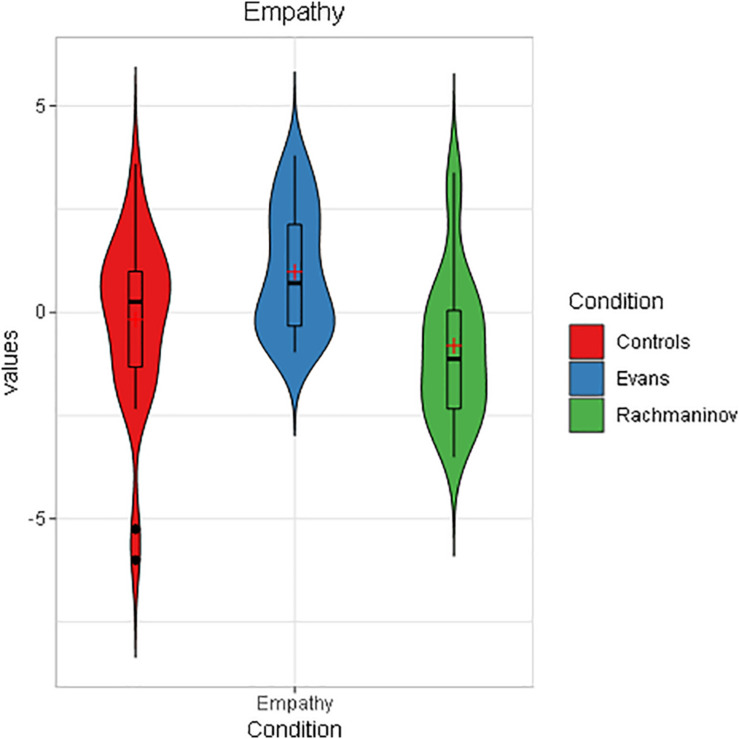
Empathy toward the character as a function of condition in Study 2 (violin plot). The boxplots within each violin represent interquartile ranges (IQRs). Red crosses indicate means, black horizontal lines indicate median, and black points are outliers. As in Study 1, participants in Evans condition showed significantly higher empathy ratings.

Results on empathy replicated those of Study 1, although the scale reliability lowered until α = 0.75. A one-way ANOVA showed a main effect of the condition, *F*(2,89) = 7.96, *p* = 0.001, $\eta_{\text{p}}^{2}$ = 0.15, (1 – β) = 0.95. Subsequent Bonferroni-corrected *post hoc* analyses revealed a significant mean difference between Evans (*M* = 0.98, *SD* = 1.49) and Rachmaninov (*M* = −0.80, *SD* = 1.77, *p* < 0.001) and Evans and Controls (*M* = −0.17, *SD* = 2.07, *p* = 0.041) (Figure 4). It is worth noting that in this study, the rating of the Rachmaninov group was much lower than in Study 1. We hypothesize that the laboratory environment, as opposed to a domestic setting, could have played a role in intensifying the anxious feeling, especially since the immersive sound quality of the laboratory audio system could have led to perceive better the peculiar low frequencies of this piece, the main carrier of the anxiety evoked.

#### Affective State Attributed to the Character

As in Study 1, results on the affective states attributed to the character were not significant (*p* > 0.05). We deepen this negative finding in Section “General Discussion.”

#### Impressions of Personality

As in Study 1, a one-way MANOVA was conducted with personality traits as dependent variables and soundtrack as the factor. A significant multivariate main effect was found for the soundtrack, *F*(10,170) = 4.40, Wilks’ Λ = 0.63, *p* < 0.001, $\eta_{\text{p}}^{2}$ = 0.21, (1–β) = 0.99.

In particular, the soundtrack significantly affected two out of five attributed personality traits (scale reliability α = 0.61) (Table 5).

**TABLE 5:** ANOVA—personality traits attributed to the character.

Personality trait	*F*	*p*	$\eta_{\text{p}}^{2}$	(1 – β)
Neuroticism	2.37	0.09	0.05	0.469
Agreeableness	6.54	<0.001	0.16	0.965
Conscientiousness	7.70	0.001	0.14	0.942
Extraversion	0.84	0.316	0.02	0.250
Openness	2.31	0.089	0.05	0.487

According to Bonferroni-corrected *post hoc* tests, in correspondence of the Rachmaninov track, the character was seen as less agreeable (*M* = 3.08, *SD* = 0.87) as opposed to the Evans group (*M* = 3.95, *SD* = 0.74, *p* = 0.008) and controls (*M* = 3.77, *SD* = 0.98, *p* < 0.001). Contrarily, in the case of conscientiousness, the Rachmaninov group showed the highest rating (*M* = 5.03, *SD* = 0.84) as opposed to the Evans group (*M* = 4.45, *SD* = 0.73, *p* = 0.02) and controls (*M* = 4.18, *SD* = 0.98, *p* = 0.001) (Table 7).

#### Plot Anticipation: Past vs. Future Perspective

**FIGURE 5:**
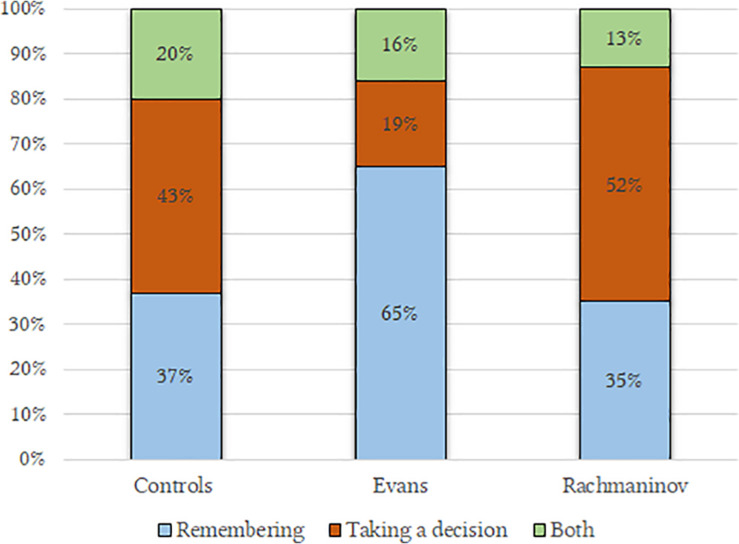
Plot anticipations as a function of the condition. Coherently with Study 1, the Evans’ track led the participants to imagine a remembering character; in contrast, half of the participants in the Rachmaninov’s group imagined a character on the point of taking a decision.

The distribution is very similar to that of Study 1, although the chi-squared test only grazed the statistical significance, possibly due to the smaller sample, χ^2^(4,92) = 8.68, *p* = 0.06, η condition dependent = 0.092 (Figure 5).

##### Emotions related to a possible character’s memory

Once again, the same pattern as in Study 1, Evans’ music led to imagine more pleasant memories (*M* = 0.19, *SD* = 0.1.00) as opposed to Rachmaninov’s track (*M* = −0.92, *SD* = 0.86) and controls (*M* = −0.04, *SD* = 0.71), *F*(2,54) = 7.61, *p* = 0.001, $\eta_{\text{p}}^{2}$ = 0.22, (1 – β) = 0.93. Subsequent Bonferroni-corrected *post hoc* comparisons revealed significant mean differences both between the two soundtracks (*p* = 0.001) and between Rachmaninov and controls (*p* = 0.022) (Table 8).

##### Moral nature of the decisions

Concerning the moral nature of the decision, we observed the same pattern of Study 1, namely the Rachmaninov group thought of very negative decisions (*M* = −0.70), followed by controls (*M* = −0.39) and Evans (*M* = −0.31); nevertheless, results did not reach the significance (*p* > 0.05), possibly due to the less numerous sample (Table 8).

#### Environment Perception

As concerns the scale reliability, we grazed a moderate/acceptable alpha (α = 0.67), we replicated Study 1 results. A one-way ANOVA revealed a main effect of the condition on the environment perception, *F*(2,89) = 5.02, *p* = 0.009, $\eta_{\text{p}}^{2}$ = 0.10, (1 – β) = 0.80. The mean difference between the two soundtracks was significant (*p* = 0.007); like in the previous experiment, Evans elicited the best evaluation of the environment (*M* = 4.21, *SD* = 0.90), followed by controls (*M* = 3.74, *SD* = 1.10) and Rachmaninov (*M* = 3.45, *SD* = 0.84) (Table 8).

#### Gender Differences

As in the previous study, gender was added in every analysis as the factor but showed no main effects nor interactions.

### Eye Tracking and Pupillometry: Methodology, Metrics, and Hypotheses

We used iMotions (iMotions) software for the stimuli presentation, connected to a Tobii X2-30 Compact (screen-based) for eye-tracking recording. Gaze data have been captured at 30 Hz.

In the vast domain of eye tracking, five main metrics may be of use for our aims: time spent, fixations, revisits, dispersion (Coutrot et al., 2012), and pupillometry.

#### Time Spent, Fixations, Revisits, and Dispersion

A fixation is a period during which our eyes are locked toward a specific point. Typically, the fixation duration is considered to be 100–300 ms. Fixations are considered a good measure of interest and visual attention in studies dealing with still images; nevertheless, given the dynamicity of our stimuli, and the contradictory evidence in the above-reported studies (*Study 2: Self-Assessment and Eye Tracking*), we preferred to use the mere time spent on a particular area of interest.

Concerning revisits, their number indicates how many times viewers returned their gaze to a particular point. Since one might revisit a spot because s/he considers it to be pleasing or confusing, revisits can be seen as a cue to the visual interest.

For the measurement of gaze points, given that the stimulus was not a still image but a movie scene, we built two moving areas of interest (mAOIs): the first one on the main character’s full body (head included) and the second on the almost hidden cameraman.

As for the dispersion, it consists of a metric apt “to estimate the variability of eye positions between observers” (Coutrot et al., 2012, p. 4). As in the here mentioned study, we defined it as follows:

$$D\left(\text{p}\right)=\frac{1}{n\left(n-1\right)}\sum_{i=1}^{n}\sum_{\begin{array}{c} j=1\\ j\neq i \end{array}}^{n}\sqrt{{\left(x_{i}-x_{j}\right)}^{2}+{\left(y_{i}-y_{j}\right)}^{2}}$$

To the best of our knowledge, it is the first time that this metric is used with the same visual stimulus accompanied by two different soundtracks and control condition.

We hypothesized that the anxiety communicated by Rachmaninov’s track would induce an enhanced state of alert, the feeling that something is about to happen; therefore, the participants in this condition will explore the space in search for possible lurking dangers. On the contrary, Evans’ track, characterized by lower arousal and a melancholic mood, might dampen the state of alertness, reducing the space exploration and promoting the focusing on the main character.

H_1_: Rachmaninov group will pay more attention to the hidden character (i.e., more time spent on his AOI and more revisits)

H_2_: Rachmaninov group will pay less attention to the main character

H_3_: Rachmaninov group will have higher dispersion ratings

#### Pupillometry

Pupillometry is considered as a reliable response system in psychophysiology. Changes in pupil size can reflect diverse cognitive and emotional states (for reviews, see Sirois and Brisson, 2014; Mathôt, 2018), ranging from arousal, interest, and effort in social decisions (Kret and Sjak-Shie, 2019). Here our focus is on arousal: we believe that the two soundtracks will elicit opposite arousal tendencies, with Rachmaninov being the most and Evans the least arousing.

We measured pupil dilation during the whole visual stimulus. Since Rachmaninov’s track induces an anxious mood, we hypothesized a greater pupil dilation in that condition.

H_4_: The Rachmaninov group will have greater pupil dilation as opposed to the Evans group.

#### Eye Tracking and Pupillometry Results

First of all, following the criteria of Breeden and Hanrahan (2017), we invalidated all recorded data in which the error rate (eye-tracker error + gaze point off-screen) for a specific participant exceeded a threshold of 10% (*N* = 3). *Time Spent, Revisits, and Dispersion* and *Pupillometry* report the analyses for time spent and revisits and for pupillometric data, respectively.

##### Time spent, revisits, and dispersion

Three one-way ANOVA analyses were conducted to verify H_1_, with the soundtrack as the factor and time spent and revisits as dependent variables. Both time spent and revisits on the hidden character were significant: we found a main effect of the soundtrack, *F*(2,86) = 3.10, *p* = 0.05, $\eta_{\text{p}}^{2}$ = 0.06, (1 – β) = 0.58 and *F*(2,86) = 3.00, *p* = 0.05, $\eta_{\text{p}}^{2}$ = 0.06, (1–β) = 0.56. Subsequent least significant difference (LSD) *post hoc* analyses revealed that for time spent (ms), the Rachmaninov group (*M* = 6,197, 15%) showed higher ratings as opposed to the Evans group (*M* = 4596, 11%, *p* = 0.04) and controls (*M* = 4,486, 11%, *p* = 0.02). As for revisits, only the mean difference of the two experimental conditions showed significance (*p* = 0.02) with Rachmaninov group’s ratings being *M* = 11.07 and Evans *M* = 8.07; that of the controls were *M* = 8.67. We can conclude that H_1_—Rachmaninov inducing more attention to the hidden character—was verified.

Nevertheless, these effects could also be due to participants making, on average, more eye movements in the Rachmaninov group and not in particular on the hidden character. To control this, we run the same analysis on an irrelevant portion of the scene (a window), the ANOVA resulted to be not significant. These results were also confirmed by an analysis on time spent and revisits made on the whole scene.

As for H_2_, concerning the attention paid to the main character, the time spent and revisits on the mAOI were not different in the three conditions.

We then analyzed dispersion^2^ to verify H_3_. An analysis of covariance (ANCOVA) with the frames as a covariate and the condition as the factor revealed a main effect of the condition, *F*(2,10157) = 132.76, *p* < 0.001, $\eta_{\text{p}}^{2}$ = 0.025, (–β) > 0.99. In greater detail, our prediction (H_3_) was not verified since the multiple comparisons *post hoc* analyses with Bonferroni correction showed that the controls (*M* = 198.09, *SD* = 54.09) had significantly higher dispersion as opposed to the Evans group (*M* = 180.24, *SD* = 52.79, *p* < 0.001) and the Rachmaninov group (*M* = 179.43, *SD* = 52.90, *p* < 0.001). Although we were unable to prove H_3_, what we found (i.e., higher dispersion in the absence of music) is not a new result in the literature (Coutrot et al., 2012), and we claim it to be explicable in terms of attention focusing (see section “General Discussion”). Lastly, an effect of the frames was found, *F*(1,10156) = 28.95, *p* < 0.001, $\eta_{\text{p}}^{2}$ = 0.003, (1 – β) > 0.99, in that the dispersion significantly varied over time.

##### Pupillometry

iMotions provides autoscaled normalized data for pupil dilation in which each value is obtained by averaging the pupil size of both eyes. The average pupil size of the first 150 ms from the stimulus onset (fade in from black) was used as a baseline for two reasons: first, dealing with a video stimulus widely changing in luminance, it was not useful to create a blank screen with the average luminance of the stimulus; second, as the music onset in each of the three conditions was after the first 150 ms and the very first screen was exactly the same in terms of luminance, screen used, and room luminosity, the three baselines could by no means differ among each other. However, to be on the safer side, the baseline fragments were not included in any of the analyses.

Following Lemercier et al. (2014), the percentage change in pupil diameter was assessed for each data point by using the following equation:

$$\%change=\frac{\left(Xdata-baseline\right)}{baseline}\times 100$$

Since the timeframe is coherent among the three conditions, we run a one-way ANOVA with the soundtrack as the factor and the percentage change of pupil diameter as the dependent variable. A main effect of the condition was found, *F*(2,5139) = 38.09, *p* < 0.001, $\eta_{\text{p}}^{2}$ = 0.01, (1 – β) > 0.99. Subsequent *post hoc* analyses using Bonferroni correction revealed two differences to be significant (*p* < 0.001 both for Evans/CC and Evans/Rachmaninov) (Table 6).

**TABLE 6:** Bonferroni *post hoc* analysis of percentage change in pupil diameter.

Condition	Condition	Mean difference	*SE*	Significance
CC (ambient sound only) (*M* = 0.27; *SD* = 0.24)	Evans (melancholic jazz)	0.070	0.008	0.000
	Rachmaninov (anxious orchestral)	0.017	0.008	0.107
Evans (melancholic jazz) (*M* = 0.20; *SD* = 0.23)	CC (ambient sound only)	–0.070	0.008	0.000
	Rachmaninov (anxious orchestral)	–0.052	0.008	0.000
Rachmaninov (anxious orchestral) (*M* = 0.25; *SD* = 0.24)	CC (ambient sound only)	–0.017	0.69	0.107
	Evans (melancholic jazz)	0.052	0.008	0.000

**FIGURE 6:**
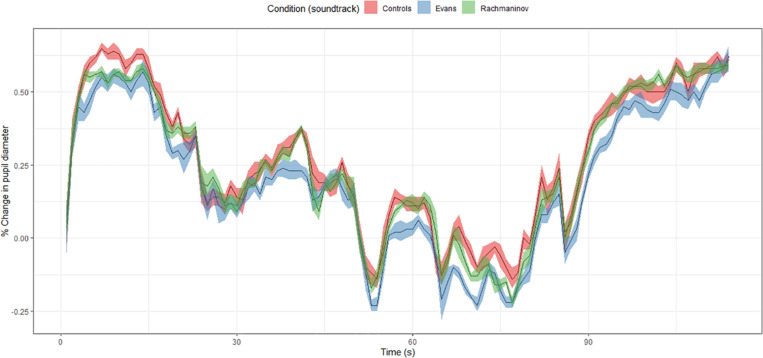
Percentage change in pupil diameter as a function of time (s) and condition. Throughout the whole scene, the pupils of the participants in the Evans group had significantly lower dilation as opposed to those in the other conditions.

As hypothesized in H_4_, the anxious soundtrack (Rachmaninov) caused greater pupil dilation (*M* = 0.27, *SD* = 0.24) as opposed to the melancholic one (Evans) (*M* = 0.20, *SD* = 0.23). Nevertheless, quite unexpectedly, the greatest pupil dilation overall was found in the controls (*M* = 0.27, *SD* = 0.24) (Figure 6). This finding contradicts previous findings by Wöllner and Hammerschmidt (2018), in which participants’ pupil diameters were larger in an audiovisual compared to a visual-only condition but confirms the findings by Batten and Smith (2018). Our result, as well of that of Batten and Smith (2018), might be also due to a strong attentional increase: while the soundtracks somehow help disambiguate the scene and frame it by providing information, the viewing of that dark and quite static scene without any music might produce a sense of hanging, an increase in the arousal and cognitive load observable through the pupil dilation.

Apparently, our data show that in control and Rachmaninov conditions, pupil dilation was higher, indicating more arousal, while in Evans, it was lower, cueing to more relaxation. We can account for such different patterns stating that the total absence of the soundtrack (or the awkward presence of silence) produced a higher state of alert, whereas the romantic/melancholic mood evoked by Evans favored a sweeter and more relaxing view.

Nonetheless, pupillometric data should always be interpreted with the caveat of their intrinsic non-specificity: pupil dilation can be due to an increase in either arousal or cognitive load (Sirois and Brisson, 2014). Unfortunately, both of these features are crucial to the interpretation process. Thereupon, it is not trivial to bear in mind that any observed difference in pupil dilation might heavily depend on the inherent characteristics of a specific visual stimulus.

### Aggregation of Self-Reports and Eye-Tracking Data

#### The Hidden Character: Did Participants Really See Him?

Participants in the Evans group answered that they had noticed the hidden character less than the others: only 48% of them responded positively (level of chance), as opposed to the Rachmaninov group (64%) and controls (73%).

Therefore, we wanted to verify through mAOIs whether the participants who said they had seen someone else actually looked at the hidden cameraman more. An alternative explanation could have been that the anxious music would have led to imagine someone else even if they had not seen him. For this purpose, we ran two one-way ANOVA analyses with the answer to the question “Have you seen someone else in the scene?” (i.e., yes/no) as the factor and time spent and revisits as dependent variables. Here, we found a main effect of the yes/no answer both for time spent, *F*(1,86) = 20.06, *p* < 0.001, $\eta_{\text{p}}^{2}$ = 0.18, (1 – β) = 0.99, and for revisits, *F*(1,86) = 10.70, *p* = 0.002, $\eta_{\text{p}}^{2}$ = 0.11, (1 – β) = 0.89; namely, regardless of the soundtrack, those who answered *yes* actually spent more time (M_Y_ = 14.95%, *SD* = 7.57; M_N_ = 8.38%, *SD* = 5.00) and revisits (M_Y_ = 10.56, *SD* = 4.96; M_N_ = 7.12, *SD* = 4.59) on the mAOI of the hidden character.

#### Does the Interpretation Change in Dependence on the Time Spent on the Main Character?

**FIGURE 7:**
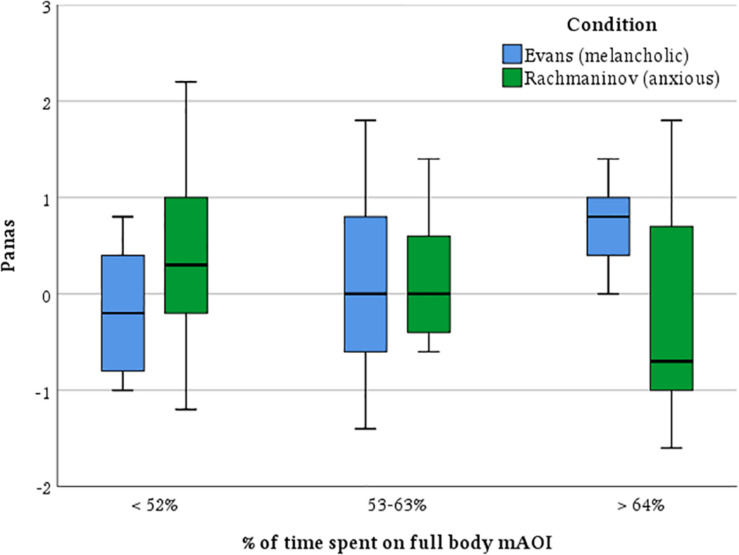
Affective state attributed to the main character as a function of the time spent on his moving area of interest (mAOI). The boxplots represent interquartile ranges (IQRs). Black horizontal lines indicate median. As the participants watched the main character to a larger extent, with Rachmaninov’s track, they progressively decreased the affective state attributed to him; conversely, with Evans’ track, they progressively increased the affective state attributed to the main character.

Our next question was, how does the scene interpretation change depending on how much the participant’s attention is focused on the main character? In order to answer this, we split our sample into three balanced subsamples based on the percentage of time spent on the main character, and we ran a two-way ANOVA with factors being the time spent on the mAOI of the main character and the soundtrack and dependent variables empathy toward the character, affective state attributed to him, and impressions of personality. No significant main effect was found, but we found a strong interaction effect for the attributed affective state, *F*(4,80) = 3.35, *p* = 0.014, $\eta_{\text{p}}^{2}$ = 0.144, (1 – β) = 0.82; namely, it seems that with the anxious music, the more our participants looked at the main character, the more negative the affective state attributed to him, whereas those who focalized more on the character with the melancholic track tended to attribute him a more positive affective state (Figure 7).

## General Discussion

Our work aimed to show the extent to which a soundtrack affects the interpretation of a short movie scene; we did it by measuring, for the first time to the best of our knowledge, several dependent variables together in a randomized fashion. The results are very satisfying.

The strongest finding concerns empathy. In both studies, we saw a substantial increase in the empathic response toward the main character in correspondence of the sweet jazz soundtrack (Empathy Toward the Character in Study 1: Online Survey and Empathy Toward the Character in Study 2: Self-Assessment and Eye Tracking) (Figures 2, 4 and Table 7), while in the other conditions, our participants had scores close to 0. As hypothesized by previous work (Pisciottano, 2019), extradiegetic music was unconsciously perceived as an emotionalizing comment on the scene, something speaking for the main character’s feelings. Evans’ music communicated sweet and melancholic feelings, paving the way to an empathic response; on the contrary, the low empathy scores of controls and Rachmaninov’s track suggest higher feelings of distress due to the unpredictable situation in the former case and a state of alert in the latter.

**TABLE 7:** Results of Study 1 and Study 2: empathy, affective state, and impressions of personality.

					Big 5				
		*N*	Empathy	Aff. State	Neuroticism	Agreeableness	Conscientiousness	Extraversion	Openness
**Study 1 (*N* = 118)**	**Rachmaninov**	46	0.21			3.98	5.61**	3.38**	
	** Evans**	37	1.16**			4.51*	4.91	2.72	
	**Controls**	35	–0.13	NS	NS	3.84*	4.82	4.82	NS
	**omnibus *p***		0.002			0.036	0.008	0.049	
	**$\eta_{\text{p}}^{2}$**		0.10			0.05	0.08	0.05	

**Study 2 (*N* = 92)**	**Rachmaninov**	31	–0.80		4.09	3.08**	5.03**		4.16
	**Evans**	31	0.98**		3.93	3.95	4.45		4.69
	**Controls**	30	–0.17	NS	3.53	3.77	4.18	NS	4.34
	**omnibus *p***		0.001		0.09	<0.001	0.001		0.08
	**$\eta_{\text{p}}^{2}$**		0.15		0.05	0.16	0.14		0.05

Bonferroni correction post hoc analyses. All the results for p < 0.10 are listed. *Only the mean difference between the two with the single asterisk is significant. **All mean differences with the other conditions are significant (p < 0.05).

As for the affective state attributed to the main character, we did not find any effect (*Affective State Attributed to the Character* in *Study 1: Online Survey* and *Affective State Attributed to the Character* in *Study 2: Self-Assessment and Eye Tracking*) (Table 7): an unexpected result, that music influenced the vast majority of our dependent variables without affecting the emotions attributed to the main character. Ultimately, emotional contents are what music is all about. We shall come back to this issue in *Conclusion*. Nevertheless, we are confident to find such an effect in further studies, possibly by using different scales that can better deepen the role of the most involved emotions.

Concerning the impressions of personality, our findings are mixed but encouraging; on the one hand, we found a multivariate effect both in Study 1 and Study 2 (*Impressions of Personality* in *Study 1: Online Survey* and *Impressions of Personality* in *Study 2: Self-Assessment and Eye Tracking*). As to main effects, things become more varied; we found significance for two personality traits in both studies (i.e., agreeableness and conscientiousness), while extraversion was significant in Study 1, and neuroticism and openness were nearly so in Study 2. It must be stressed that in both studies, the most affected traits were the ones related with relational and moral aspects more than mere psychological features: as for the high agreeableness, this attribution might be considered a precursor to empathy; in fact, in both studies we found a correlation between them, *r*(118) = 0.36, *p* < 0.001 and *r*(92) = 0.46, *p* < 0.001. Overall, the data suggest that the Evans music increased the character’s agreeableness, something similar to what was found by Hoeckner et al. (2011), where the likeability of a character increased with melodramatic music as opposed to thriller music or no music. On the contrary, Rachmaninov decreased agreeableness, while in parallel, it increased the character’s perceived conscientiousness, possibly due to the sense of austere and serious atmosphere evoked by the lowest notes of double basses and cellos.

The results show that music also influenced plot anticipations, confirming other findings (Bullerjahn and Güldenring, 1994; Vitouch, 2001; Shevy, 2007); in both our studies, Evans led our participants to imagine a character who was remembering more than deciding something i.e., embracing a past perspective. In particular, the memories associated with this music were characterized as slightly pleasant whereas Rachmaninov let our participants think of someone planning morally bad deeds (*Moral Nature of the Decisions*) (Table 8).

**TABLE 8:** Results of Study 1 and Study 2: plot anticipations and environment perception.

		Plot anticipation					
		Remembering	Taking a decision	Emotions of memories	Moral nature of decisions	Environment perception
**Study 1 (*N* = 118)**	**Rachmaninov**	39%		39%	−0.70*	0.01*	3.67*
	**Evans**	57%		11%	0.10*	1.21*	4.34*
	**Controls**	43%		37%	–0.23	0.25	4.17
	**omnibus *p***		0.05		0.017	0.011	0.025
	**$\eta_{\text{p}}^{2}$**				0.09	0.13	0.067

**Study 1 (*N* = 92)**	**Rachmaninov**	35%		52%	−0.92**		3.45*
	**Evans**	65%		19%	0.19		4.21*
	**Controls**	37%		43%	–0.04	NS	3.74
	**omnibus *p***		0.06		0.001		0.009
	**$\eta_{\text{p}}^{2}$**				0.22		0.10

Bonferroni correction post hoc analyses. All the results for p < 0.10 are listed. *Only the mean difference between the two with the single asterisk is significant. **All mean differences with the other conditions are significant (p < 0.05).

Concerning environment perception, in both studies, Evans music made it appear (*Environment Perception* in *Study 1: Online Survey* and in *Environment Perception* in *Study 2: Self-Assessment and Eye Tracking*) (Table 8) more interesting, pleasant, likable, warmer, and safer, like in similar results already found in very different contexts (Michon and Chebat, 2004; Yamasaki et al., 2015). This result confirms the holistic nature of the interpretation process, showing that music, a human artifact, extends its capacity of shaping not only on the narratives involving human features (emotions, feelings, plans, etc.) but also on inanimate objects and the environment of the scene. It would be very reductive to say that music only expresses feelings: much more than that, it actually shapes our view.

Finally, eye-tracking data of Study 2 provide new physiological insights about scene viewing: music can improve focusing (*Time Spent, Revisits, and Dispersion*), as found by Auer et al. (2012) and Mera and Stumpf (2014), and also influences pupil dilation (*Pupillometry*). Unfortunately, no consistent evidence can be claimed on this matter, since the only similar study (Wöllner and Hammerschmidt, 2018) found opposite results, namely pupil diameters larger in the audiovisual (both with tranquil and dogged music) compared to the visual-only condition. We might explain such conflicting evidence as follows: since two out of the three clips used by those authors depict very dynamic actions (a beating and a chase), participants would not need music to be aroused, and soundtrack worked as an *associative enhancer* (Brown, 2006) in an already-arousing situation. Conversely, in our control condition, silence could have worked as an enhancer of arousal by eliciting a sense of hanging (*Time Spent, Revisits, and Dispersion*), making the pattern of this and other dependent variables often overlap with that of the anxious condition. Furthermore, coherently with Coutrot et al. (2012), our control condition reported the highest ratings in dispersion, confirming the role of music in the attention focusing process throughout a visual scene.

## Limitations

Despite its satisfying results, this study has some relevant limitation: first of all, the two pieces differ from each other not only in terms of their emotional content but also in their genre. Moreover, one is a solo performance, while the other is fully orchestral. Strictly speaking, we cannot be sure whether such differences could have affected the interpretations; we should have at least created two more conditions: one with a solo/anxious piece and another with an orchestral/melancholic one searching for genre or quantity of instruments effects. Undoubtedly, several other finer-grained manipulations could have been performed; we could have manipulated the mode only of the same melody: major vs. minor, as in Ziv and Goshen (2006), or degree of dissonance, as in Bravo (2013). Nevertheless, as a first attempt, we opted for two tracks that were radically dissimilar so to be able to discover also moderate effects with a relatively small sample.

Another concern lies in the absence of a listening-only condition in which the participants had to imagine a scene instead of watching it; this could have been profitable to understand the extent to which the semantic musical information was related to the music itself or to the associative processes with the visual material. We plan to use a similar design in further studies.

Finally, we are aware that these results heavily depend on the semantic content of the visual scene: in the current study, to be sure enough to find noticeable effects of the soundtrack, given the intrinsic primacy of the video contents on the audio ones when making sense of an audiovisual, we opted for a semantically weak scene in which no concrete action was done. We are confident that as the visual content becomes stronger, the power of the music becomes weaker, shifting from a decisive role to an ancillary one. Furthermore, as the visual becomes stronger, we hypothesize that there is an increase in the search for the congruity with the soundtrack, possibly leading to stronger and more coherent contents when satisfied or highly memorable and uncanny scenes when unsatisfied. For instance, who does not remember Hannibal Lecter butchering the guards to the soft (and diegetic) sound of Johann Sebastian Bach’s *Aria da Capo*? (BWV 988)? Or Alex DeLarge and his droogs singing *Singin’ in the Rain* in the famous raping scene of *A Clockwork Orange*?

## Conclusion

Every time we confront a new stimulus—an event, a news, a novel, a piece of music, a poem—we need to interpret it, that is, to make sense of it: to understand it and integrate it into our previous knowledge base by connecting new and old information through inferences and logical relations. To do so, we need to add information to the stimulus, whether on our own or thanks to an “interpreter.” A judge in his verdict interprets the rule by shaping it onto a specific transgression; a pianist interprets a Sonata by adding his expressivity; a historian allows us to better interpret the present conflict between two neighboring countries by telling of their history. Information may be added either on a top–down or a bottom–up basis (Shevy, 2007). In the former case, it is provided by our point of view: on a deeper level by our goals or previous knowledge, our personality, and memories; on a more surface level by our contingent affective and cognitive state. Such a point of view superimposes a frame to the stimulus, like sand molds shape the sand or like colored lenses give a color to the world we see. In the bottom–up case, information is drawn from outside: we may better comprehend a poem or a philosophical essay, thanks to a literary critic or an exegetical comment. However, if nobody else hands us such information, given our incessant need, as humans, to search for knowledge everywhere, we can figure out some, filling in the gaps of the unsaid by our inference and imagination. For instance, to understand the plot of a novel or a movie, we need to know the characters’ goals and personalities, and if we have no information whatsoever, we try to take advantage of any cue to guess them. More specifically, we need to build narratives that must be coherent among the involved modalities, therefore audio and video in our case, as in Cohen’s (2013) Congruence-Association Model.

In our studies on how music affects a visual scene interpretation, this is what seems to happen in our participants. Viewing quite an ambiguous scene and needing to make sense of it, they resort to whatever contextual information, and they profoundly rely on music. If the background music is sweet and melancholic, they either feel (in the emotion-induction hypothesis, Juslin and Laukka, 2004) or cognitively represent (in the representation hypothesis, Brown, 2006) a sad feeling that paves the way for an empathic feeling toward and makes the participants attribute him sad memories. Conversely, if the music is disquieting, attributing this to a sense of alert and evil intentions, they do not feel empathy toward him. In this way, participants can build a coherent and interconnected structure of the scene and expectancies about its plot. This is how we might integrate the results of our studies: in fact, although we have found that multiple variables are affected by the soundtrack, we do not know what are the relationships among them. We plan to investigate their specific intertwining and causal chain in future studies.

In any case, our work provides new evidence of the extent to which music can shape the interpretation of a scene; by measuring several dependent variables entailed by this process (empathy toward a character, attributed affective states and impressions of personality, plot anticipations, environment perception), we came up with a multidimensional perspective of scene interpretation. Robust effects emerged on empathy, plot anticipations, environment perception, and two impressions of personality (agreeableness and conscientiousness) were heavily influenced by music, while the three others (neuroticism, extraversion, openness) less so. Taken together, these results indicate that the soundtrack had a greater impact on some attributed dimensions that are more stable and personal (i.e., impressions of personality and empathic feeling) compared to the contingent affective state; indeed, we may account for this result by recurring to the actor–observer bias (Jones, 1972), according to which people tend to judge their own behavior relying on contingent factors, while the others’ actions as caused by dispositional aspects. Surely, further studies need to prove this point, but it looks plausible that when anticipating or judging a character’s behavior, viewers would be exposed to such a bias, namely, they tend to overattribute causality to the character’s personality (i.e., dispositional factors) while avoiding to explain motivations by situational factors as the contingent affective state.

These studies represent an attempt to fill in the theoretical and methodological gaps reported by Herget’s (2019) review. Although our results seem very promising, further investigations are needed. Here, the two musical excerpts differed from one another in terms of several musical features; having found solid effects with such tracks, the further challenge might be to employ subtler manipulations on specific musical cues already proved to be linked to emotional expression (Eerola et al., 2013), such as mode (Ziv and Goshen, 2006), tempo (Fernández-Sotos et al., 2016), dynamics, and timbre (Hailstone et al., 2009; Wu et al., 2014). Another topic to explore is what it means that a music piece *fits* a visual scene; something similar has been proposed for audiovisual advertising (Herget et al., 2018), but one might deepen what are the effects provoked by music that does not fit a scene and whether this can elicit cognitive dissonance.

Furthermore, being the political campaigns advertising conceived as proper audiovisual contents (Killmeier and Christiansen, 2004; Ezell, 2012; Shevy and Hung, 2013), it would be profitable to analyze how audio (speech and background music)-visual fitness can affect the message appeal in terms of credibility and voting intentions.

## Data Availability Statement

The raw data supporting the conclusions of this article will be made available by the authors, without undue reservation.

## Ethics Statement

The studies involving human participants were reviewed and approved by Commissione Etica of Roma Tre University. Written informed consent for participation was not required for this study in accordance with the national legislation and the institutional requirements.

## Author Contributions

AA conceived and designed the study and wrote the manuscript. AA and MM collected the data, organized the database, and performed the statistical analyses. FD’E supervised the analysis. IP contributed in the conclusion and supervised the whole work. All authors contributed to manuscript revision, read and approved the submitted version.

## Conflict of Interest

The authors declare that the research was conducted in the absence of any commercial or financial relationships that could be construed as a potential conflict of interest.

## Footnotes

1 An antiballot box stuffing was employed in order to avoid multiple participations from the same device.

2 Given that we located each gaze point by means of Cartesian coordinates overlapping the screen resolution (0≤x≤1920 | 0≤y≤1080), dispersion values are given in pixels.
